# Risk factors and mortality among newborns with persistent pulmonary hypertension

**DOI:** 10.12669/pjms.295.3728

**Published:** 2013

**Authors:** Athar Razzaq, Ahmed Iqbal Quddusi, Naila Nizami

**Affiliations:** 1 Dr. Athar Razzaq,MBBS, FCPS, Trainee Fellow in Neonatal Pediatrics, The Children Hospital &Institute of Child Health, Multan, Pakistan.; 2 Dr. Ahmed Iqbal Quddusi,MBBS, FCPS, Head of Neonatal Pediatrics, Warden’s House, Rafia Hall, Girls Hostel, Nishter Medical College, Multan, Pakistan.; 3 Dr. Naila Nizami,MBBS, FCPS, Trainee Fellow in Neonatal Pediatrics, The Children Hospital &Institute of Child Health, Multan, Pakistan.

**Keywords:** Persistent pulmonary hypertension, Birth asphyxia, Meconium aspiration syndrome

## Abstract

***Objective: ***To determine the risk factors for persistent pulmonary hypertension of newborns (PPHN) and their influence on mortality.

***Methods: ***This was an observational study conducted at The Children’s Hospital & the Institute of Child Health, Multan, Pakistan, from July 2011 to June 2012.All admitted babies who had respiratory distress, cyanosis and evidence of hypoxia on ABG,s were diagnosed provided that they were having right- to- left or bidirectional hemodynamic shunting at the ductus arteriosus or at patent foramen ovale along with Tricuspid regurgitation (TR) jet >40 mm of Hg on echocardiography. All the demographic, maternal, antenatal, natal and postnatal data were recorded on a predesigned Performa.

***Results: ***There were 79 patients, including 61 males and 18 females. The most common risk factors observed in our study were male sex (72.1%), cesarean section mode of delivery (54.2%), positive pressure ventilation while resuscitation (44.2%) birth asphyxia (40.4%) and meconium aspiration syndrome (MAS)35.4%. It was found that male sex (88.8%), cesarean-section delivery (77.7%), respiratory distress syndrome (RDS) 44.8% and sepsis (44.4%) were more associated with PPHN in premature infants than with term and post term infants. Out of the total 79 patients, death occurred among 7 preterm and 14 terms and post term infants. As a whole, cesarean section mode of delivery (71.4%), birth asphyxia (57.1%) and female sex (52.4%) were found major risk factors associated with mortality. However, respiratory distress syndrome (Relative Risk RR=5), birth asphyxia (RR=2.5) and male sex (RR=2)were found to be associated with increased risk of mortality in preterm than term and post term infants.

***Conclusion:*** Male gender, cesarean section mode of delivery, MAS and RDS are the major risk factors for PPHN in any age group. RDS, Birth asphyxia and male sex are associated with increased risk of mortality in pre term than term and post term infants.

## INTRODUCTION

Persistent pulmonary hypertension of the newborn (PPHN) is a clinical syndrome that results from failure of normal fetal- to- neonatal circulatory transition and is associated with substantial neonatal morbidity and mortality.^1^Although persistent pulmonary hypertension is less common, but more significant cause of respiratory distress in newborns than others like transient tachypnea of newborn, respiratory distress syndrome and etc. An incidence is of 1.9 per 1000 live-births (0.4–6.8/1000 live births) have been reported, and mortality rate ranging from 4–33%have been reported.^2^

Pulmonary hypertension is normal and necessary state for the fetus. As the placenta, not the lungs, serve as the organ of gas exchange. Most of the right ventricular output crosses the ductus arteriosus to the aorta, and only 5-10% of the combined ventricular output is directed to the pulmonary vascular bed. This high pulmonary vascular tone in fetus is due to increased pulmonary vasoconstrictors like low oxygen tension, endothelin-1, leukotriene’s and Rho kinase, and decreased vasodilators like prostacyclin and nitric oxide (NO).^3^ Normally in the fetus and early neonatal life, there is extra pulmonary shunting across patent foramen ovale and ductus arteriosus because of elevated pulmonary vascular pressures.^4^

In some newborns, the normal decrease in pulmonary vascular tone does not occur and eventually results in PPHN. With inadequate pulmonary perfusion, neonates are at risk for developing refractory hypoxemia, respiratory distress, and acidosis.^5^ PPHN is most often recognized in term or near-term neonates, but it can occur, albeit infrequently, in premature neonates. Despite the introduction of treatment with drugs like sildenafil, prostacyclin, nitric oxide, extracorporeal membrane oxygenation and advanced modes of mechanical ventilation, about 4 to 33% of the affected infants still die and those who survive may suffer from serious and long term sequelaes like chronic lung disease, seizures and neurodevelopmental problems.^6^^,^^7^

There are many established perinatal risk factors for developing PPHN like post maturity, nonvertex presentation, fetal distress, cesarean section, meconium staining of the amniotic fluid or aspiration, neonatal sepsis, and pneumonia. Other risk factors like low maternal education; black ethnicity; tobacco use, fevers, urinary tract infection, pulmonary disease, vaginal bleeding and maternal diabetes are considered inconsistent by few studies.^8^

PPHN is a disease of serious nature and increased mortality and its risk factors like birth asphyxia, MAS and sepsis are quite frequent in our setups. During the literature research for local studies, it was found that limited data are available about PPHN, thus aim of this study was to see the relationship between the persistent pulmonary hypertension of the newborn and its risk factors and detect their role in mortality in our practice.

## METHODS

This observational study was conducted at Neonatal Intensive Care Unit of The Children’s Hospital &The Institute of Child Health, Multan, Pakistan, over a period of 12 months from July 2011 to June 2012.The study was approved by the Institutional Medical Ethics Committee and informed consent was obtained from each parent.

All the babies who were admitted with gestational age > 34 weeks (which was defined from their mother^’^s last menstrual period) having respiratory distress and showing profound hypoxemia (PaO_2 _<50mm of Hg) on their arterial blood gas (ABG) were selected for echocardiography, performed by an experienced Pediatric Cardiologist. Among those who underwent echocardiography, neonates were designated as having PPHN who met the following two criteria.

1. Right- to- left or bidirectional hemodynamic shunting at the ductus arteriosus or at patent foramen ovale,
2. Tricuspid regurgitation jet pressure of >40 mm of Hg.

Although, pulmonary artery pressure directly can be measured by Swan Ganz Catheter. There are a number of echocardiographic indicators that help indirectly to measure pulmonary artery pressure (PAP). However, we chose peak velocity of the tricuspid regurgitation (TR) jet of more than 40 mm of Hg as one of our selection criteria. Tricuspid regurgitation jet pressure is an indicator of the right ventricular systolic pressure which is equivalent to pulmonaryartery systolic pressure after adding right atrial (RA) pressure provided that there is no pulmonary valvular obstruction as well.^9^

PAP = TR +RA

 The right atrial (RA) is generally assumed to be 5-10 mm of Hg in infants. Once diagnosed as patients of PPHN, they were categorized into mild, moderate and severe PPHN by measuring their TR between 40 to 50, 50 to 70 and > 70 mm of Hg respectively. Exclusion criteria were evidence of any congenital heart disease except for patent ductus arteriosus; patent foramen ovale; atrial septal defect; or a single, small muscular venticuloseptal defect and newborns with other congenital abnormalities.

Various demographic characteristics (e.g., name), the mother^’^s medical history (e.g., maternal diabetes, pulmonary disease, idiopathic hypertension), pregnancy conditions (e.g., pregnancy induced hypertension and use of drugs etc.), fetal distress, mode of delivery, need of resuscitation, clinical evidence of hypoxsic ischemic encephalopathy supported by birth history, neonatal lung diseases (e.g., meconium aspiration syndrome, respiratory distress syndrome, etc.),were recorded for all the babies (Table-I). The newborns with PPHN were stratified into late pre term (born after 34 but less than 37 weeks of gestation), term (from 37 weeks of gestation to 42) and post term infants (>42weeks of gestation) to control the confounding variables.

All newborns were managed according to our unit protocols designed for PPHN. The outcome of the babies was noted as being dead or survived (at discharge time) and various risk factors affecting morality were determined and compared between pre term and term/post term infant groups as shown in Table-II.

**Operational Definition**

***Fetal Distress:*** Fetal distress was labeled in only those newborns that had history provided by their Obstetricians of decrease fetal movement, meconium stained liquor, fetal tachycardia or bradycardia during or after a contraction, decreased variability in the fetal heart rate and late decelerations.

***Birth Asphyxia***: The diagnosis of birth asphyxia was made mainly on the basis of clinical data collected through suggestive history i.e. delayed cry, prolonged labor pain , clinical examination (consistent with hypoxic ischemic encephalopathy) and relevant investigations i.e. ABG’s, Cranial USG, LFT, Renal function tests, Echocardiography, LDH , CPK & Urine examination (hemoglobinuria) etc.

***Maternal Diabetes***: If the mother had history of fasting plasma glucose level >126 mg /dl or a casual plasma glucose >200 mg /dl whether before or during pregnancy.

***Maternal Hypertension***: It was defined as blood pressure elevation to 140 mm of Hg systolic or 90 mm of Hg diastolic over two measurements at least 6 hours apart. 

***Sepsis:*** The diagnosis of neonatal sepsis was made on the basis of history, clinical examination and also with the help of laboratory investigations i.e. blood culture, CBC with platelet count, CRP, Toxic granulation, CSF & Urine examination and culture and etc.

***Meconium Aspiration Syndrome:*** Those newborns having respiratory distress along with meconium staining of vocal cords, umbilical cord or nails were diagnosed as cases of meconium aspiration syndrome

***Respiratory Distress Syndrome:*** RDS was diagnosed on clinical history and examination and also with the help of X-ray chest and ABG’s.

All the information was entered in a pre designed Performa. Social Package of Statistical Science (SPSS version 19) was applied for the final analysis.

## RESULTS

During the study period of a year, we enrolled 79(57 males and 22 females) infants with the diagnosis of persistent pulmonary hypertension of newborn. All were referred cases as our hospital does not have maternity services. Of the 79 patients, 18 were preterm (born after 34 but less than 37 weeks of gestation), 61term and post term (>37 weeks of gestation).

Table-I present clinical characteristics of infants with PPHN. As a whole, male sex (72.1%), cesarean section mode of delivery (54.2%), positive pressure ventilation while resuscitation (44.2%) and birth asphyxia (40.5%) were observed major risk factors for developing PPHN. Compared with the term and post term, pre term infants with male sex (88.8%), born by cesarean-section (77.7%), complicated by respiratory distress syndrome (44.4%) and sepsis (44.4%) were relatively more associated with PPHN. Of the total 79 patients, 58(73.4%) survived and death resulted in 21 patients (26.6%), as shown in Table-II. This table also gives comparison of the different risk factors associated with death between pre term and term & post term infants in the form of Relative Risks (RR).

This was an observational study and data was summarized as counts, percentages and relative risks using SPSS version 19 for final analysis.

**Table-I:** Selected perinatal risk factors and characteristics of the newborns with PPHN

***Characteristics***	***Over all*** ***N =(79) (n%)***	***Preterm34-37 wks.*** ***N=18 (n%)***	***Term& Post term*** ***37 wks. and above*** ***N=61 (n%)***
*Gender of babies*			
Male Female	57 (72.1) 22 (27.9)	16 (88.8) 2 (11.2)	41 (67.2) 20 (32.8)
*Mod of delivery *			
SVD^*^ C-section^**^	36 (45. 6) 43 (54.2 )	4 (22.2) 14 (77.8)	32 (52.4) 29(47.6)
*Need for resuscitation*			
Not needed Only oxygen PPV^***^	21 (26.5) 23 (29.3) 35 (44.2)	7 (38.9) 7 (38.9) 4 (22.2)	14 (22.9) 16(26.2) 31 (50.9)
*Fetal distress*	11 (13.9)	3 (16.6)	8 (13.1)
*Birth asphyxia*	32(40.5)	9(50)	23((37.7)
*Maternal DM*	4(5)	2(11.1)	2(3.3)
*Maternal HTN*	3(3.8)	0	3(5)
*Meconium stained liquor*	6(7.5)	0	6(9.8)
*Lung disorders*			
MAS RDS	28(35.4) 11(13.9)	2(11.1) 8(44.4)	26(42.6) 3(4.9)
*Systemic disorders*			
Sepsis	23(29.1)	8(44.4)	15(18.6)
*Echocardiography*			
Mild PPHN Moderate PPHN Severe PPHN	11(13.9) 23(29.1) 45(57)	5(27.7) 2 (11.1) 11(61.1)	6(9.8) 21(34.4) 34(55.8)
*Maximum respiratory support*			
Oxygen with mask CPAP^∞^ IMV^®^	12(15.1) 51(64.5) 16(20.4)	3(16.6) 14(77.7) 1(5.5)	9(14.7) 37(60.6) 15(24.7)

* Spontaneous vaginal delivery,

** Cesarean section,

*** Positive Pressure Ventilation,

∞ Continuous positive airway pressure,

®Intermittent mandatory ventilation

**Table-II:** Selected perinatal risk factors influencing mortality between pre term and term &post term newborns with PPHN

***Characteristics***	***Over all*** ***N =(21) (n%)***	***Preterm*** ***34-37 wks.*** ***N=7 (n%)***	***Term& Post term*** ***37 and ≥ 37 wks.*** ***N=14 (n%)***	***Relative Risk*** ***(RR)***
*Gender of babies*				
Male Female	10(47.6) 11 (52.4)	5(71.4) 2(28.6)	5(35.7) 9(64.3)	2 0.43
*Mod of delivery *				
SVD C-section	6(28.6) 15(71.4)	2(28.6) 5(71.4)	4(28.6) 10(71.4)	1 1
*Need for resuscitation*				
Not needed Only oxygen PPV	7(33.3) 6(28.6) 8(38.1)	2(28.6) 2(28.6) 3(42.8)	5(35.7) 4(28.6) 5(28.6)	0.8 1 1.5
*Fetal distress*	6(28.6)	2(28.6)	4(28.6)	1
*Birth asphyxia *	12(57.1)	2(28.6)	10(71.4)	2.5
*Lung disorders*				
MAS RDS	10(47.6)) 7(33.3)	0 5(71.4)	10(71.4) 2(14.2)	0 5
*Systemic disorders*				
Sepsis	10(47.6)	5(71.4)	5(35.7)	2
*Need for resuscitation*				
Mild PPHN Moderate PPHN Severe PPHN	6(28.5) 5(23.8) 10(47.6)	2(28.5) 2(28.6) 1(14.3)	4(28.5) 3(21.4) 9(64.3)	1 1.3 0.22
***Maximum respiratory support ***				
Oxygen with mask CPAP IMV	3(14.2) 3(14.2) 15(71.6)	0 2(28.5) 5(71.4)	3(21.4) 0 11(74.5)	0 0 0.9

## DISCUSSION

Persistence of pulmonary hypertension leading to respiratory failure in the neonate has been recognized for last 44 years. It was first described by Gersony and colleagues in 1969.^6^Since then, a number of risk factors have been attributed for this serious cause of respiratory failure in newborn infants.

It has been reported by numerous authors that there is high incidence of respiratory distress syndrome and PPHN when cesarean sections including elective caesareans were performed before 39 weeks of gestation.^10^ In normal labor; there is increased release of endogenous prostaglandins and catecholamine’s. These substances along with physical compression from birth canal result in increased clearance of lung fluid, which is not in the case of cesarean section delivery. Fetal distress, a well-known risk factor for PPHN is the major reason for cesarean section as well. Therefore, the increased incidence of PPHN after cesarean section might be largely attributable to underlying fetal conditions that triggered the intervention and ultimately resulting in PPHN, rather than to a direct causal effect of cesarean section (or lack of vaginal delivery) per se. Our findings also include a high rate of PPHN following elective cesarean delivery particularly in preterms, and it suggests that treating Obstetricians should consider this added morbidity when performing cesareans. In another study from California, USA by Winovitch KC and colleagues also reported the similar findings, so, this mode of delivery along with higher incidence of RDS and sepsis probably puts the preterm infants at a greater risk of developing PPHN.^11^

The transition from fetal to neonatal life is a dramatic and complex process involving extensive physiologic changes at birth. It is estimated that approximately 5% to 10% of all births will require some form of resuscitation beyond basic care.^12^In our study, a large number of neonates that required positive pressure ventilation during resuscitation were associated with PPHN. It was found that those who required such interventions had some sort of intrauterine hypoxia. Hypoxia and acidaemia, both of which are powerful pulmonary vasoconstrictors prevent the normal postnatal changes in circulation. Thus all those factors that lead to intrauterine hypoxia are responsible for developing PPHN and increased need for positive pressure ventilation at birth as well.

The first intestinal discharge from newborns is meconium and factors that promote the passage in utero include placental insufficiency, maternal hypertension etc. In our study, we found 26 newborns with meconium aspiration syndrome, majority belonging to term and post term infants. Only two pre term infants were observed with meconium aspiration syndrome. Although, the incidence of meconium aspiration syndrome is less in premature, but because of small sample size in our cohort, its exact relationship with the gestational age needs larger survey. Moreover, it’s the fetal distress that is responsible for meconium stained liquor and meconium aspiration syndrome, thus avoidance of factors resulting in fetal distress and eventually birth asphyxia might be the key to decrease the incidence of PPHN among the newborns with MAS, as observed by two other studies done in Taiwan.^8^^,^^13^

In all age groups, increased mortality was observed in newborns associated with cesarean section mode of deliveries, birth asphyxia, female sex and severe PPHN. However, when mortality was compared between pre terms and terms & post terms, it was found that for preterm infants RDS, birth asphyxia, male sex and sepsis are non protective whilst female sex, no active need for resuscitation at birth and severe PPHN are protective risk factors. However, we did not find significant difference in mortality between these groups for risk factors like mode of delivery, oxygen use during resuscitation, and with mild or moderate PPHN.

With an estimated 298,000 neonatal deaths annually and a reported neonatal mortality rate of 49 Per 1000 live births, Pakistan accounts for 7% of global neonatal deaths. Along with Infection and prematurity, birth asphyxia accounts for 87% of neonatal deaths worldwide as a whole.^14^ Birth asphyxia is an important co-morbid condition in PPHN. Considering the PPHN- related mortality; we observed this more among term and post term infants with birth asphyxia than pre terms (RR 0.4). However, a potential limitation of our study regarding this factor is the clinical based criteria to diagnose this condition like delayed cry and subsequent encephalopathy (which could have other etiologies).

Marked risk of mortality was found in premature with PPHN if simultaneously affected with RDS than other group. Deficient synthesis or release of surfactant, together with small respiratory units and a compliant chest wall, produces atelectasis and eventually results into hypoxia in RDS. Decreased lung compliance, small tidal volumes, increased physiological dead space and insufficient alveolar ventilation eventually result in hypercapnia. The combination of hypercapnia, hypoxia and acidosis produces pulmonary arterial vasoconstriction with increased right-to-left shunting through the foramen ovale and ductus arteriosus and within the lung itself and these are the reasons for PPHN in preterm with RDS. Walther and his colleagues had also found increased risk of morbidity & mortality in premature infants affected with RDS.^15^

Premature infants having PPHN, when simultaneously affected with sepsis were found to have increased risk of mortality than term and post term infants. High contribution to mortality by sepsis is well documented in developing countries owing to multiple contributing factors such as cross-infection, over-crowding and staff related negligence. As we know that premature infants are more susceptible to get sepsis,^16^^,^^17^ this puts the pre term infants to have increased risk of mortality when sepsis is accompanied with PPHN. Thus, avoidance of all those factors which enhances the chances of both early and late onset sepsis may decrease the mortality in newborns particularly in premature infants.

## CONCLUSION

In general, male gender, cesarean section mode of delivery, use of PPV while resuscitation and birth asphyxia are the major risk factors for PPHN in our study. RDS, birth asphyxia and sepsis were responsible for increased mortality particularly in pre matures having PPHN.
